# Relationship between inflammatory markers and coronary slow flow in type 2 diabetic patients

**DOI:** 10.1186/s12872-023-03275-y

**Published:** 2023-05-09

**Authors:** Moataz Ali Hasan Ali Elsanan, Islam Hussein Hassan Hussein Tahoon, Ghada Ibrahim Mohamed, Shimaa Gamal ZeinElabdeen, Islam Elsayed Shehata

**Affiliations:** Department of Cardiology, Faculty of Medicine, Zagazig University, Zagazig, 44519 Sharkia Governorate Egypt

**Keywords:** Diabetes type 2, Coronary slow flow, Inflammatory markers (NLR, PLR)

## Abstract

**Background:**

Diabetes is a serious and quickly expanding global health problem. Cardiovascular disease is the leading cause of mortality in type 2 diabetes mellitus (T2DM) patients. Coronary slow flow (CSF) is characterised by delayed distal perfusion during coronary angiography with normal coronary arteries.

This study aimed to investigate the correlation between CSF and inflammatory markers regarding glycemic status in T2DM**.**

**Methods:**

This cross-sectional study included 120 patients who were divided equally into 4 groups according to their glycemic control and presence or absence of coronary slow flow: Group I included patients with T2DM with good glycemic control without CSF; Group II included patients with T2DM with good glycemic control and CSF; Group III included patients with T2DM with poor glycemic control without CSF; and Group IV included patients with T2DM with poor glycemic control and CSF. The neutrophil-to-lymphocyte ratio (NLR), platelet-to-lymphocyte ratio (PLR), C-reactive protein (CRP), platelets, hematocrit, and haemoglobin were also evaluated as risk factors for coronary slow flow.

**Results:**

This study showed that body mass index (BMI), hematocrit level, NLR, and CRP demonstrated a moderate but significant correlation (*r* = 0.53) with CSF in poorly controlled T2DM. NLR cutoff > 2.1 could predict CSF in poorly controlled T2DM with a modest sensitivity and specificity. A 1.9 increase in HbA1c increases the likelihood of coronary slow flow. Dylipidemia increases the likelihood of coronary slow flow by 0.18 times. Other predictors for coronary slow flow include NLR, PLR, CRP, platelets, hematocrit, and hemoglobin. The effect of the predictors is still statistically significant after being adjusted for glycemic status, age, and sex (*p*** < **0.001).

**Conclusions:**

Poor glycemic control increases the incidence of CSF. This supports the hypothesis that CSF is related to endothelial dysfunction as poor glycemic control causes endothelial dysfunction due to inflammation.

**Trial registration:**

ZU-IRB#9419–3-4–2022 Registered 3 April 2022, email. IRB_123@medicine.zu.edu.eg.

## Introduction

In 2019, the International Diabetes Federation (IDF) predicted that Egypt ranks ninth among the world’s countries, with around 8,850,400 cases and a frequency of 15.2% in adults. Egypt is expected to be the seventh-largest country in the world by 2045 [1].

Coronary heart disease (CHD), heart failure, arrhythmias, and sudden cardiac death are examples of macrovascular complications. Cerebrovascular disease and peripheral artery disease are both types of cardiovascular disease in T2DM. Complications contribute significantly to morbidity and death in both types of diabetes. Cardiovascular disease is the leading cause of mortality in T2DM patients [2, 3].

Glycemic control is evaluated by haemoglobin A1c (HbA1c), continuous glucose monitoring (CGM) with either time in range (TIR) and/or glucose management indicator (GMI), and blood glucose monitoring (BGM) [4].

One of the major causes of atherosclerosis is diabetes. Inflammation has been clearly established to have a significant role in the initiation, development, and progression of atherosclerosis. CSF was a marker of endothelial activation and inflammation. HbA1c is also a significant inflammatory marker [5].

Inflammation has a role in many cardiovascular diseases, and inflammatory pathways have been recognised in the setting of CSF. Other inflammatory indicators, such as red cell distribution width and serum uric acid levels, have also been linked to the presence of CSF. Inflammatory parameter abnormalities may be a sign of endothelial dysfunction, both of which lead to CSF [6].

Therefore, the current study aimed to investigate the correlation between CSF and inflammatory markers regarding glycemic status in T2DM**.**

## Materials and methods

We enrolled 120 diabetic patients of type 2 with chronic coronary syndrome (CCS) at our Cardiology Department, Faculty of Medicine, Zagazig University, Egypt.

### Inclusion criteria

All T2DM patients who were referred for coronary angiography and had coronary slow flow were included in the study. The presence of typical chest discomfort or positive or conflicting results from noninvasive screening measures for myocardial ischemia served as the justification for coronary angiography. If non-invasive assessment indicates high risk occurrences, coronary angiography is recommended. The CSF is basically defined as a delay in the progression of the contrast that is injected into the coronary arteries during coronary angiography [6].

### Exclusion criteria

Patients with valvular heart disease, atrial fibrillation, cardiomyopathies, congenital heart disease and hematological disorders were excluded.

### Study population

This study included all patients with T2DM on conventional glycemic control (CGC); Admission blood glucose, 180–200 mg/dl) by conventional therapy using insulin and Dipeptidyl-peptidase 4 (DPP4) inhibitors who referred for coronary angiography who also had CSF. The patients were divided into four groups based on their glycemic control and the presence or absence of CSF. Group I: patients with T2DM with good glycemic control without CSF (*n* = 30); Group II: patients with T2DM with good glycemic control and CSF (*n* = 30); Group III: patients with T2DM with poor glycemic control without CSF (*n* = 30); Group IV: patients with T2DM with poor glycemic control and CSF (*n* = 30). NLR was measured in each group to see how it related to glycemic control parameters and predicted the occurrence of CSF. Other predictors of the occurrence of CSF were also investigated.

### Clinical assessment

#### Complete history taking

Age, gender, and CAD risk factors such as hypertension, diabetes, smoking, dyslipidemia, and a family history of premature coronary artery disease (CAD) are all considered.

According to the ESH/ESC Guidelines for the Management of Arterial Hypertension, hypertension is defined as blood pressure levels of 140 mmHg SBP and/or 90 mmHg DBP [7].

Diabetes mellitus was diagnosed on basis listed by American Diabetes Association (2010) as: Fasting blood sugar ≥ 126 mg/dl or 2 h postprandial blood sugar ≥ 200 mg/dl or HBA1C ≥ 6.5 or Symptoms of diabetes plus casual plasma glucose concentration ≥ 200 mg/dl. Casual is defined as any time of day without regard to time since last meal. The classic symptoms of diabetes include polyuria, polydipsia, and unexplained weight loss [8].

Smoking was defined as active smoking in the last 6 months [9].

Dyslipidemia was considered according to recommendations of Third Report of the National Cholesterol Education Program (NCEP), when any of the following was present: serum cholesterol ≥ 200 mg/dl, LDL ≥ 100 mg/dl, HDL < 40 mg/dl for high-risk patients [10].

Family history of premature CAD, defined by the presence of at least a first degree relative with a cardiovascular event or premature SCD at a young age (< 65 years for women and < 55 years for men) [11].

#### General and local examination

Blood pressure, BMI, and electrocardiogram (ECG) were measured to all patients. Body Mass Index (BMI), defined as the body weight divided by the square of the body height in meters, and is universally expressed in units of kg/m^2^, resulting from mass in kilograms and height in meters [12].

#### Invasive coronary angiography

The diagnostic criteria for CSF are based on the recommendations of Beltrame [13]. At coronary angiography, there was no coronary artery stenosis or stenosis 40%, and when the delay of distal angiographic contrast agent filling reaches a corrected TIMI frame count (cTFC) value greater than 27 frames (30 frames/s), at least one coronary artery is involved.

#### Transthoracic echocardiography (TTE)

**Fig. 1:**
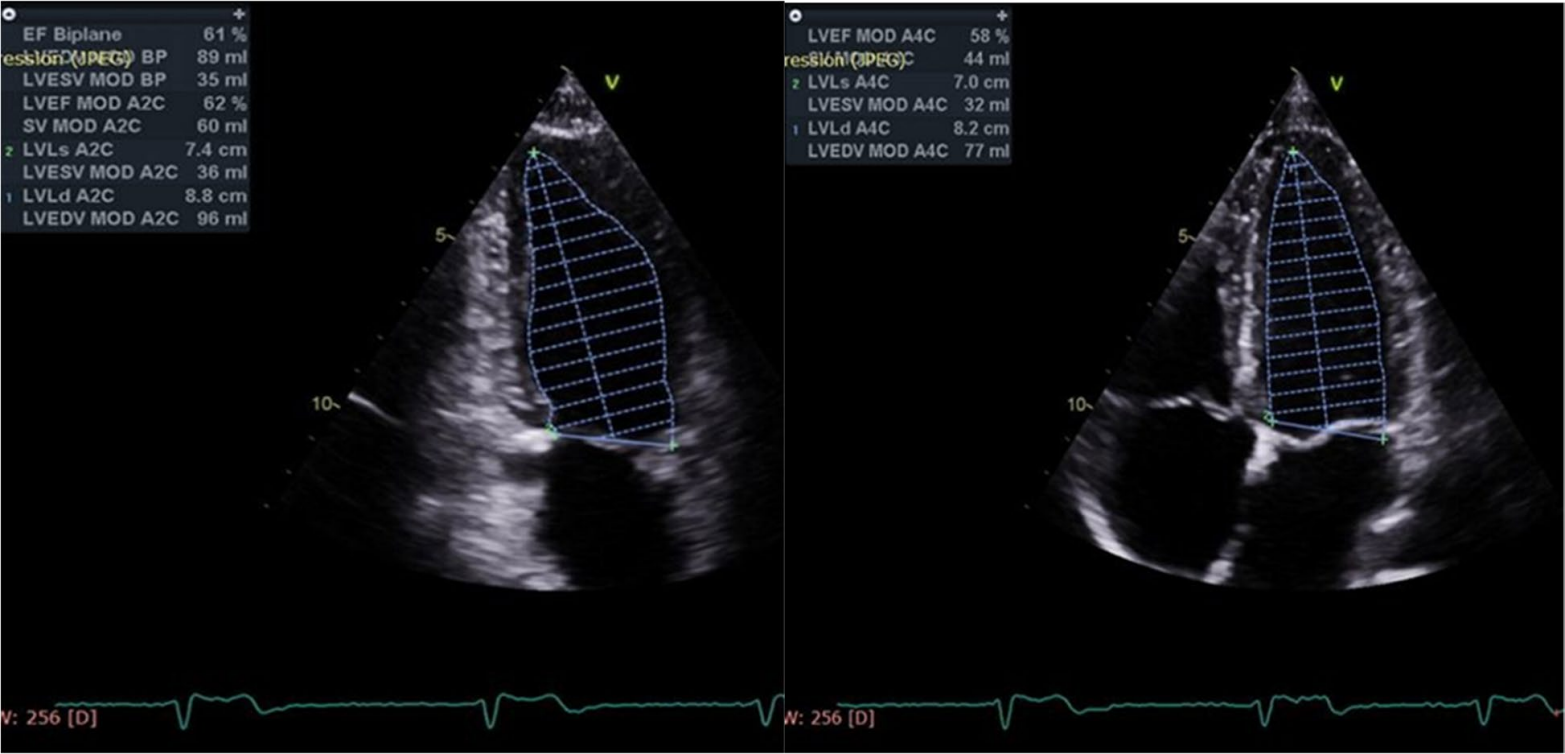
EF Simpson’s Method: EF (61%), LVESV: (35 ml) and LVEDV: (89 ml)

All patients underwent TTE using the commercially available system (Vivid E9, General Electric, Horten, Norway, 2013) on admission for the coronary angiography procedure. Standard images were obtained using a 3.5–5 MHz transducer at a depth of 16 cm in the parasternal and apical views. The TTE study was performed according to the recommendations of the American Society of Echocardiography and European Association of Echocardiography, and measurements were indexed to body surface area when indicated. Assessment of resting regional wall motion abnormalities, wall motion score index (WMSI), ejection fraction and LV end-diastolic and end-systolic volumes were calculated using the biplane Simpson’s method and resting wall motion abnormalities (Fig. 1) [14].

#### Laboratory investigations

Blood samples of the patients were obtained in the morning between 8:00 a.m. and 10:00 a.m. after a fast of at least 8 h. The blood samples of all patients obtained for complete blood count (CBC), fasting plasma glucose, HbA1c, and hsCRP were studied. The NLR of the patients is calculated by dividing the absolute neutrophil number by the absolute lymphocyte number. The neutrophil-to-lymphocyte ratio (NLR), platelet-to-lymphocyte ratio (PLR), C-reactive protein (CRP), platelets, hematocrit, and haemoglobin were also evaluated as risk factors for coronary slow flow.

### Statistical analysis

Version 26 of the Statistical Program for Social Science (SPSS) was used to analyse the data**.** Continuous data were represented as mean and SD, and categorical data were represented as event and percentage. One-way ANOVA, independent t-test, chi-square test, or exact Fisher test, and Pearson correlation were used. ROC curve analysis and the Kappa test were used to detect the ideal cut-off values of the predictors with the greatest sensitivity and specificity. When the probability of error is less than 5% (*p* < 0.05), the result is significant.

## Results

This study revealed that there was no statistically significant difference between groups in terms of age, gender, or CAD family history.

This investigation revealed that there was no statistically significant difference in haemoglobin levels across groups.

Hematocrit, platelet lymphocyte ratio (PLR), and c-reactive protein (CRP) were significantly lower in patients without CSF (Group I, Group III) compared to those with CSF (Group II, Group IV) (*p*_3_, *p*_5_, *p*_6_ < 0.05) (Table 1).

**Table 1:** Laboratory assessment of included patients

	**Group I (*N* = 30)**	**Group II (*N* = 30)**	**Group III (*N* = 30)**	**Group IV (*N* = 30)**	***p*-value**
HbA1c	5.8 ± 0.2	6.1 ± 0.4	9.3 ± 0.7	9.6 ± 1	*p*_2_, *p*_3_, *p*_4_, *p*_5_, < 0.05 *p*_1,_ *p*_6_ > 0.05
FBS	104 ± 6.2	110 ± 8.5	252.6 ± 42	271.3 ± 53.1	*p*_1_ = 0.6 *p*_2_, *p*_3_, *p*_4_, *p*_5_, *p*_6_ = 0.05
Hemoglobin	12.83 ± 0.46	12.86 ± 0.49	12.82 ± 0.5	12.59 ± 0.49	0.29
Hematocrit	39.1 ± 0.7	42.2 ± 2.5	39.5 ± 1.0	42.4 ± 2.0	*p*_2_, *p*_5_ > 0.05 *p*_1_, *p*_3_, *p*_4,_ *p*_6_ = 0.05
Platelets	219.7 ± 14.6	256.9 ± 19.5	211 ± 22.1	213.3 ± 21.1	*p*_2,_ *p*_3_, *p*_6_ > 0.05 *p*_1_, *p*_4_,* p*_5_ < 0.05
Leukocytes	7.8 ± 1.5	8.5 ± 1.4	8.1 ± 1.3	9.3 ± 1.1	*p*_2_,* p*_4_ > 0.05 *p*_1_, *p*_3_, *p*_5_, *p*_6_ < 0.05
Neutrophil	3.6 ± 0.6	4.6 ± 1.9	4.4 ± 1.3	6.5 ± 1.6	*p*_4_ > 0.05 *p*_1_, *p*_2_, *p*_3_, *p*_5,_ *p*_6_ < 0.05
Lymphocyte	2.0 ± 0.4	1.9 ± 1.0	2.1 ± 0.6	1.7 ± 0.6	0.215
PLR	119 ± 33.2	150.2 ± 38.6	107.3 ± 33.8	140.7 ± 58	*p*_1,_ *p*_3_, *p*_4,_ *p*_5_, *p*_6_ < 0.05 *p*_2_ > 0.05
NLR	2.0 ± 0.6	2.6 ± 1.3	2.3 ± 1.1	4.3 ± 2.1	*p*_3_, *p*_5_, *p*_6_ < 0.05 *p*_1_, *p*_2_, *p*_4_ > 0.05
CRP	4.2 ± 0.9	7.8 ± 1.1	4.2 ± 1.6	7.9 ± 1.1	*p*_1,_ *p*_3_, *p*_4,_ *p*_6_ < 0.05 *p*_2_ > 0.05

*HbA1c* hemoglobin A1c, *FBS* fasting blood sugar, *PLR* Platelet lymphocyte ratio, *CRP* c-reactive protein, *NLR* Neutrophil lymphocyte ratio, *p*_1_ indicate the difference between Group I and Group II, *p*_2_ indicate the difference between Group I and Group III, *p*_3_ indicate the difference between Group I and Group IV, *p*_4_ indicate the difference between Group II and Group III, *p*_5_ indicate the difference between Group II and Group IV, *p*_6_ indicate the difference between Group III and Group IV

CSF correlates positively with NLR in patients with inadequate glucose management (Table 2).

**Table 2:** Correlation between Coronary slow flow and biomarkers regarding glycemic status

	**Glycemic control**			
	**Good glycemic control (HBA1c < 7)**		**Poor glycemic control (HBA1c > 7)**	
	***r***	***p*-value**	***r***	***p*-value**
**NLR**	-0.011	0.924	0.548^**^	** < 0.001**
**PLR**	0.530^**^	** < 0.001**	-0.042	0.800
**CRP**	0.885^**^	** < 0.001**	0.509^**^	**0.001**
**Hemoglobin**	0.148	0.186	0.382^*^	**0.018**
**Platelets**	0.746^**^	** < 0.001**	-0.022	0.897
**Hematocrit**	0.501^**^	** < 0.001**	0.542^**^	** < 0.001**

*NLR* Neutrophil-to- lymphocyte ratio, *PLR* Platelet-to-lymphocyte ratio, *CRP* C-reactive protein

**Fig. 2:**
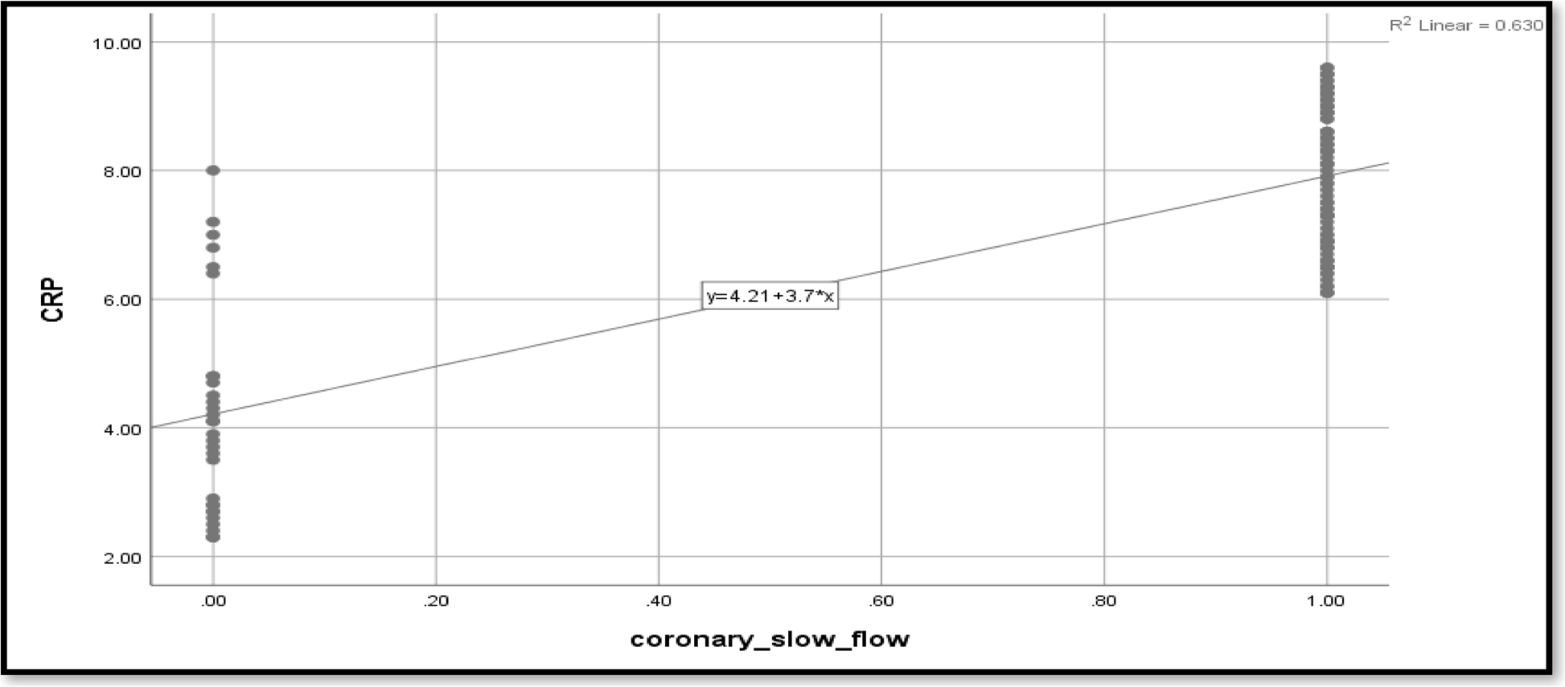
CRP as a predictor for coronary slow flow

**Fig. 3:**
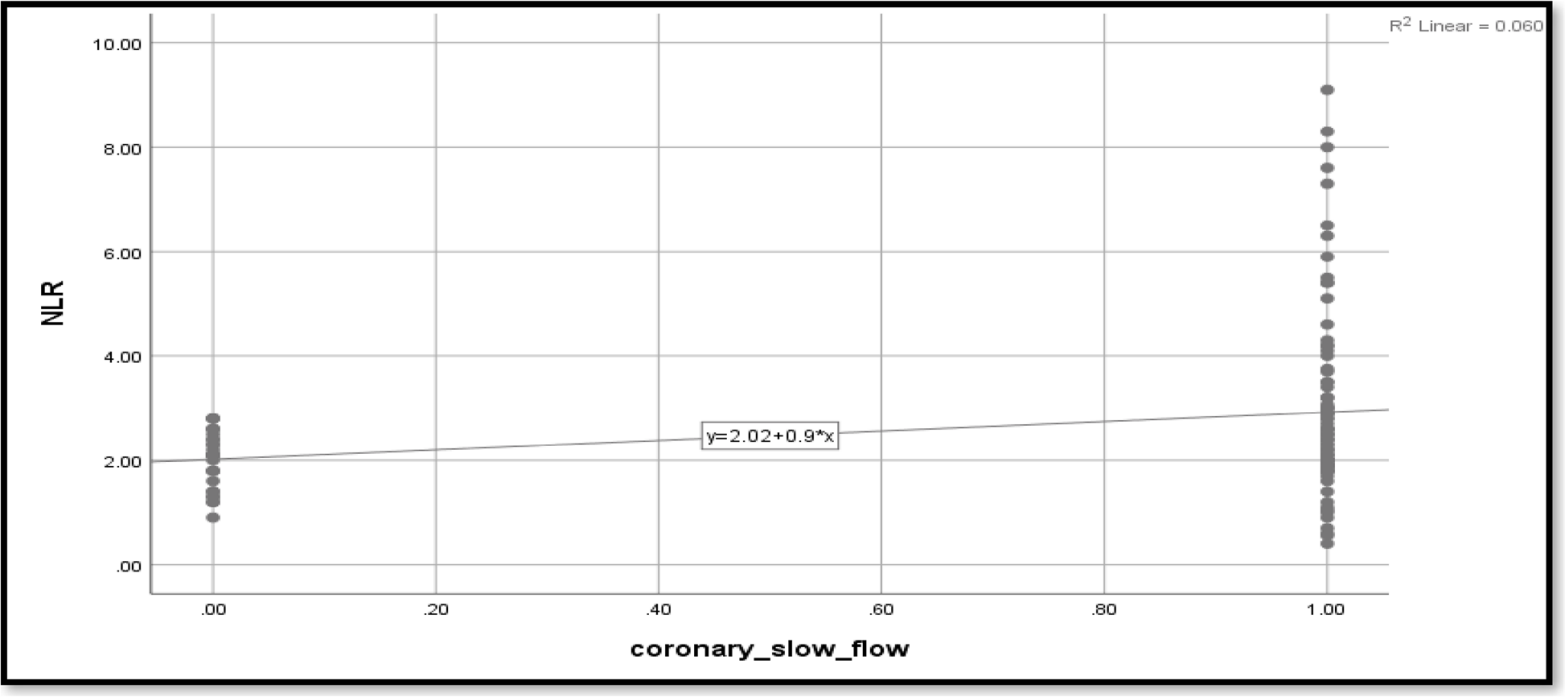
NLR as a predictor for coronary slow flow

**Fig. 4:**
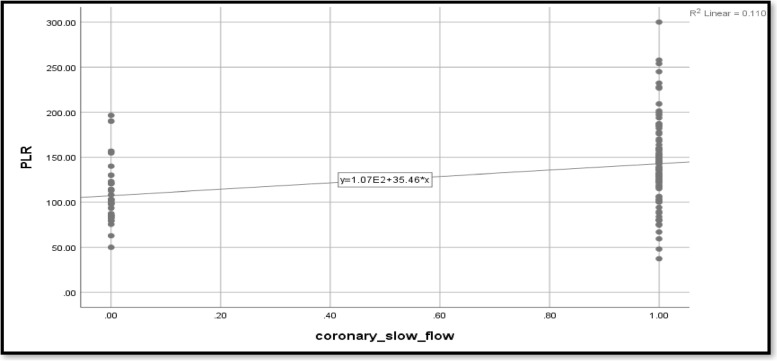
PLR as a predictor for coronary slow flow

The current study looked at the relationship between coronary slow flow and NLR in relation to glycemic status. Coronary slow flow shows a moderately significant correlation with NLR (*r* = 0.548) (Table 3). When compared to non-hypertensive patients, the presence of hypertension increases the likelihood of coronary slow flow by 4.66 times. Smoking causes 3.5 times as many cases of coronary slow flow as nonsmokers. Dyslipidemia increases the likelihood of coronary slow flow by 0.18 times. Other predictors for coronary slow flow include NLR, PLR, CRP, platelets, hematocrit, and hemoglobin. The effect of the predictors was still statistically significant after being adjusted for glycemic status, age, and sex (*p* < 0.0001) (Table 3; Figs. 2, 3 and 4).

**Table 3:** Predictors for coronary slow flow

	**Crude odds ratio (OR)**			**Adjusted odds ratio (aOR**^**a**^**)**		
	**OR**	**95% CI**	***p*-value**	**OR**	**95% CI**	***p*-value**
**Age**	1.000	0.953–1.049	0.990	–––-	–––-	–––-
**Sex (female)**	1.144	0.498–2.631	0.751	–––-	–––-	–––-
**Hypertension**	4.667	1.906–11.426	**0.001**	4.638	1.892–11.36	**0.001**
**Smoking**	3.500	1.44–8.5	**0.006**	3.516	1.4–8.559	**0.006**
**BMI**	7.599	2.92–19.714	** < 0.001**	7.31	2.83–18.877	** < 0.001**
**Dyslipidemia**	0.182	0.075–0.443	** < 0.001**	0.177	0.072–0.434	** < 0.001**
**Family history**	0.821	0.305–2.213	0.697	–––-	–––-	–––-
**CRP**	6.413	2.827–14.545	** < 0.001**	14.220	3.7–54.005	** < 0.001**
**NLR**	1.864	1.149–3.023	**0.012**	1.896	1.166–3.083	**0.010**
**PLR**	1.023	1.01–1.036	**0.001**	1.023	1.010–1.036	**0.001**
**Lymphocyte**	0.820	0.509–1.320	0.413	–––-	–––-	–––-
**Neutrophil**	1.299	0.967–1.744	0.082	–––-	–––-	–––-
**Leukocytes**	1.261	0.916–1.735	0.155	–––-	–––-	–––-
**Platelets**	1.04	1.023–1.058	** < 0.001**	1.065	1.038–1.093	** < 0.001**
**Hematocrit**	2.456	1.673–3.607	** < 0.001**	2.454	1.670–3.607	** < 0.001**
**Hemoglobin**	2.972	1.172–7.540	**0.022**	2.990	1.178–7.590	**0.021**
**WMSI**	0.643	0.150–2.746	0.551	–––-	–––-	–––-
**Ejection fraction**	1.031	0.943–1.128	0.503	–––-	–––-	–––-
**ECG**	1.249	0.546–2.859	0.598	–––-	–––-	–––-

*BMI* Body mass index, *CRP* C-reactive protein, *NLR* Neutrophil-to-lymphocyte ratio, *PLR* Platelet-to-lymphocyte ratio, *WMSI* Wall motion score index, *ECG* Electrocardiogram

^a^Adjusted for glycemic status, age, sex

## Discussion

Inflammation plays a role as an important pathogenic factor for various cardiovascular diseases, along with coronary heart disease. So, the neutrophil-to-lymphocyte ratio (NLR) has been reported to affect various inflammatory diseases, including some cardiovascular diseases. NLR is also an important factor in cardiovascular diseases in terms of morbidity and mortality [6].

In patients with T2DM and CSF, our study showed that NLR had a moderately significant correlation with glycemic control parameters. The study performed by Sefil et al. [15] reported results that were similar to those found in this study. Hussain et al. [16] reported similar results between NLR and glycemic regulation. They found a significant association between NLR and HbA1c among the groups divided into three according to their glycemic control status. In this study, NLR values after treatment were not investigated. Which explained by presence of DM can induce inflammation and release of inflammatory markers which induce endothelial dysfunction.

In a prospective study on a non-diabetic 38,074-strong cohort, an average of 6-year follow-up NLR was associated with the incidence and prevalence of T2DM. This finding suggests that the NLR is a predictor of diabetes development [17].

Our study found no significant relationship between NLR and glycemic control parameters in patients with T2DM who did not have CSF. This agreement with Mendes et al. [18] showed that hyperglycemic subjects had a NLR similar to that of normoglycemic subjects but had a lower PLR.

Our study showed that NLR was higher in patients with CSF than in patients without CSF, especially in patients with poor glycemic control; this agrees with Cetin et al. [19]; Oylumlu et al. [20]; and Challa et al. [21]. This may explain the role of inflammation in the pathogenesis of CSF.

Despite the studies mentioned above, the association between CSF and inflammation remains unclear due to the limited number of patients in these studies and conflicting data. In a study established by Altun et al. [22], they found no significant difference between patients with CSF and patients without CSF regarding NLR.

Our study showed that poor glycemic control increases the incidence of CSF. A one-degree increase in FBS increases the likelihood of CSF by 2.4. The increase in HbA1c increases the incidence of CSF by 1.9. This can ensure the hypothesis that coronary slow flow is caused by endothelial dysfunction, as chronic hyperglycemia is well known to accelerate the development of endothelial dysfunction and the pathological process of atherosclerosis, resulting in more diffuse coronary artery lesions and worse clinical outcomes [23].

### Study limitations

The current study was restricted by its modest sample size (*n* = 120), as it was a single-center investigation. Patients with type 2 diabetes were the only ones included. All diabetic individuals should be included in future trials. Our research did not include those on chronic medication. It's possible that the long-term use of medicine contributes to the onset of coronary sluggish flow. Therefore, additional long-term studies are suggested to examine the connection between medications and CSF occurrence, preferably including the medication of patients.

### Clinical implication

Patients are more likely to develop coronary sluggish flow if they have risk factors such as (hypertension, smoking, increased BMI, dyslipidemia, high platelet count, high hematocrit value, high NLR, high PLR, and high CRP level).

## Conclusion

Poor glycemic control increases the incidence of CSF. This support the hypothesis that CSF is related to endothelial dysfunction as poor glycemic control cause endothelial dysfunction.

Other factors (hypertension, smoking, increased BMI, decreased dyslipidemia and high platelet count, hematocrit, PLR and CRP) significantly increase in patients with CSF and can be used as predictors of CSF.

## Recommendations

Further large caliber, multicenter studies are needed to consolidate our findings. Further long-term studies which include patient medication are recommended to evaluate the relation between drugs and CSF occurrence. NLR may be useful as an easily measurable, non-invasive and cost-effective parameter for follow up of glycemic control in patients with CSF and also for prediction of CSF. Good glycemic control is mandatory as poor glycemic control leads to various cardiovascular events.

## Data Availability

Our data used to support the findings of this study are available from the corresponding author upon request.

## Abbreviations

BMI: Body mass index
CSF: Coronary slow flow
T2DM: Type 2 diabetes mellitus
CRP: C-reactive protien
NLR: Neutrophil-to-lymphocyte ratio
PLR: Platelet-to-lymphocyte ratio
WMSI: Wall motion score index
CHD: Coronary heart disease
IDF: International Diabetes Federation
HbA1c: Hemoglobin A1c
FBS: Fasting blood sugar
CGM: Continuous glucose monitoring
TIR: Time in range
GMI: Glucose management indicator
BGM: Blood glucose monitoring
CGC: Conventional glycemic control
DPP4: Dipeptidyl-peptidase 4
CAD: Coronary artery disease
SBP: Systolic blood pressure
DBP: Diastolic blood pressure
LDL: Low density lipoprotin
HDL: High density lipoprotin
SCD: Sudden cardiac death
ECG: Electrocardiogram
BMI: Body mass index
NCEP: National cholesterol education program
CBC: Complete blood picture
